# The Place of Reading in the Training-School

**Published:** 1919-10-18

**Authors:** 


### THE MATRONS' AND SISTERS' DEPARTMENT.

### THE PLACE OF READING IN THE TRAINING-SCHOOL.

To find pleasure in books is not to find favour in the eyes of the old-fashioned sister. She can never watch with patience the spectacle of a probationer immersed in a book. The sight of a text-book, especially just before an examination, she may tolerate. But unless an unprofessional work be strictly utilitarian, a cook-book, for instance, or a Bradshaw, she is instantly moved to find "something better to do" for the unlucky probationer whose head is bent over it.

What place can the art, or perhaps we should say the habit, of reading take in the training-school? It is a little unfortunate that modern methods of teaching lean so much to exposition by word of mouth that few probationers appear to be able to "take in" instruction through the vehicle of print. To have to rectify the errors of the school teacher is very hard on the sister-tutor. But no probationer who does not learn how to master a few simple columns of print relating to nursing matters without having them elaborately explained to her by word of mouth can be considered well prepared for her future. It is part of her training to learn how to uncover the frame work, as it were, detect what is essential and grasp the argument. Once the art of reading intelligently even an article in the nursing or medical press has been acquired, the nurse has her advancement in her own hands, for even if opportunities for post-graduate study are altogether lacking she can keep abreast of the times. Nursing, like medicine and surgery is still to make. There are no complete manuals of its mysteries. And unless the nurse has learned how to go on studying for herself, she will early find herself imperfectly equipped.

But reading is a necessary part of the nurse's training quite apart from the purely professional point of view. The three or four years spent in hospital influence a woman's whole life and often determine whether or no she becomes a reader. Truth to tell the scale is far too often weighed down against the habit of reading, and the nurse imbibes a settled conviction that the life she has chosen gives her no time to read. Can nothing be done to set nurses free from this obsession? It is one which tells grievously against them. It shuts them from both the world of art and letters, from the drama of modern history unfolded before their eyes, from the refreshment of poetry, from the free current of ideas which animates those with whom they are brought into contact, from many intimate joys, from the sweetest and most easily accessible of all forms of recreation. Let us make at least an effort to remove from our training-schools the stigma of discouraging the love of books.

It is not always remembered that the first step towards encouraging people to read is to provide them with books. It is an unfortunate fact that the library in many institutions is a disgrace. There will be a few shelvesful of books, purchased or presented years ago without much discrimination, perhaps grown grey in the service of successive probationers. The collection is padded out with ancient medical treatises and odd volumes of magazines, and shows a little tattered group of seven-penny novels as its most promising ornament.

The remedy is a subscription among the nurses to some circulating library of good repute where twenty volumes at a time can be had on very easy terms, especially if books published within the last six months are not expected. With a good background of standard works in the library, and an average of of say twenty circulating books—fiction, travel, biography, art, etc.—to each forty nurses, all could be well served. A little trouble taken by one of the sisters as librarian to order acceptable works, and see that everyone had a volume which suited their tastes, would ensure that the books were well used. Any popular or striking work could be made the occasion for a little symposium. Books would become one of the topics of the day, and when particular interest centred round one about which every one was talking it would get engaged long beforehand. A well understood rule that all probationers were expected to have an extra-professional book on hand would do much.

It may seem unduly optimistic to expect nurses in general to love poetry, but it may safely be said that all who do not lack intelligence can be led to admire what is brought to their notice with zest by another reader. It has soothed and comforted so many nurses in silent watchings to have passages from the poets at command that it seems worth while to try whether the habit of repeating verse cannot be stimulated among us. Occasionally a meeting might take place at which those whose names were drawn by ballot were expected to recite some favourite lines or short poem. And a somewhat similar entertainment proves very popular, at which all whose names are drawn read a chosen passage (not more than five minutes) from a favourite book, with a word of explanation if necessary. Such meetings are well adapted to local centres where indeed much can be done to stimulate a love of books.

